# The Hygiene of Gestation

**Published:** 1871-12

**Authors:** John Stainback Wilson

**Affiliations:** Atlanta, Georgia


### THE HYGIENE OF GESTATION.

*Abstract of a Paper Read before the Atlanta Academy of Medicine, being a part of the Report from the Section on Hygiene.*

BY JNO. STAINBACK WILSON, M.D., ATLANTA, GEORGIA.

All will admit that pregnancy is a natural, physiological condition, and that there is nothing incompatible with its existence and the enjoyment of perfect health. Yet, every physician is called on daily to prescribe for some one of the formidable list of the "diseases of pregnancy," which have become so common, in the present unphysiological mode of living, as to cause a general belief among the people that pregnancy is nothing less than a *nine months' sickness.* And so it *is* with most women, from ignorance or neglect of the plain precepts of hygiene. Consequently, the child-bearing life of woman—by far the most important period of her existence—is embittered by numberless diseases; her health is destroyed, and she becomes incapable of performing her conjugal and maternal duties, either through physical inability or through disgust and aversion, arising from a dread of the sufferings to which she has been previously subjected in gestation and parturition. Hence, the obligations of maternity are abhorred and evaded; marital rights and enjoyments are denied; the happiness of wedded life is blighted, and woman, bereft of the fascinating charms which cluster around her as wife and mother, sinks into cold indifference and moping melancholy, willing to sacrifice the happiness of her husband, and even to destroy the fruit of her own body, rather than to discharge the sacred and Heaven-enjoined obligations of maternity. How important, then, is the hygiene of gestation, when it is known that most of the diseases of pregnancy, and the dangers and miscalled *accidents* of parturition, arise from violations of the laws of health!

The importance of this subject might be urged and enforced, again, by considering the influence of the mother over the child *in utero.* But on this comprehensive theme I can only say, that the transmission of intellectual, moral and physical peculiarities or conditions from parents to children, is one of the best-established facts in physiology. This great truth is to be read in family resemblances, in hereditary diseases, and in mental and moral traits of character. It is also admitted, that of the two parents, the mother has, for obvious reasons, the greater modifying power over the elements of the new being. And it should never be forgotten that the transmitting and modifying power of parents embraces not only *original* and congenital qualities or peculiarities—regarded heretofore as alone transmissible—but that acquired qualities, the results of bad habits, may be thus transmitted.

If, then, the offspring is composed of the *very essence* of each parent *as he or she is actually constituted at the time of conception*, and if the mother has such a controlling modifying power over the child *in utero*, by virtue of her intimate connection with it, the importance of placing her under the most favorable circumstances, at the time of conception and during gestation, cannot be well over-estimated or exaggerated.

Let us, then, as briefly as possible, consider the hygienic means best calculated to secure, during gestation, that perfection of health and that integrity of function which are necessary to the proper performance of the act, and to guard against the deplorable evils to which allusion has been made.

I will state, as an incontrovertible fact, and as a guiding principle in this discussion, *that pregnancy, though a natural condition, is one of excitement.*

If any proof of this is needed, it is to be found in the vast changes and the extensive sympathies of the organ of gestation. The containing power of the womb is increased more than *five hundred times;* and, of course, this vast change in an organ which is the very centre of sympathetic excitement in woman—in an organ which *makes her what she is*—arouses every function of the body into activity. This is seen in the increased frequency and force of the pulse—in the quicker breathing—in the higher temperature—in the greater activity of the urinary and cutaneous secretions—in the greater alertness of the mind, and in the exaltation and ready impressibility of the nervous sensibilities. In short, the whole system is in a state of unusual activity—*in a state of excitement bor-*

*dering on fever.* Yet the condition is a normal physiological one, such as belongs to the state of pregnancy. It is by no means to be regarded as a disease, but should be made our guide in adopting measures suited to the condition.

Viewing the condition of pregnancy as one of excitement predisposing to inflammatory diseases, I think we will have no difficulty in deciding what kind of FOOD is best suited for a pregnant woman. As to the *quantity* of food, it is not surprising that the people should have the idea that a pregnant woman must have an extra allowance of food, because "she has to eat for two." But *we* know the quantity of food is to be measured by the digestive powers, and that any excess beyond these powers is a source of irritation and debility, rather than of support and strength. The rule, then, should be: *Eat so much as the stomach can easily manage, and no more.*

As to the kind of food, it may be premised that every well-arranged dietetic system should consist in a due combination of albuminous, oleaginous and farinaceous constituents. The albuminous are required when an unusual amount of nervo-muscular exertion is put forth. And this class of elements is most advantageously derived from animal flesh. The oleaginous elements are needed for the sustentation of heat, and are to be obtained equally from the vegetable and animal kingdoms. But a larger proportion of the farinaceous elements, as a substitute for the oleaginous, is most favorable to health under a high atmosperic temperature.—*Dr. Carpenter.*

Now, taking the above in connection with the activity of all the functions in pregnancy, and especially the increased evolution of heat, there can be but little difficulty in deciding the diet best adapted to the condition.

Though gestation is not attended with any unusual nervo-muscular *exertion*, as expressed by Carpenter, yet even larger drafts are made on both the nervous and muscular systems, by the demands of pregnancy, than could be required for any ordinary or even extraordinary muscular exertion. Therefore, the food, in this condition, should be highly nutritive, abounding sufficiently in histogenetic elements for the support of mother and child.

Now, while animal food abounds more in histogenetic elements than vegetable food, the former contains more of the hydrocarbons—more heat-generating elements; and its free use is, therefore, contraindicated by the general excitability of the system and the special activity of calorification in pregnancy.

Again: the too free use of animal food increases unduly the amount of nutritive and effete material, thus causing over-fullness of the vascular system, already over-excited in the early stages of pregnancy by sympathy, and oppressed in the latter stages by pressure on the heart and great blood-vessels. Thus will we have hemorrhages, dropsical effusions, etc.; or the functions of the depurating organs will be over-taxed to rid the system of the excess which has been thrown into the blood.

It is hardly necessary to say that this state of things is likely to give rise to organic affections of the lungs, kidneys, and other depurating organs—affections far more serious than the immediate effects of repletion.

Another objection to animal food is, the want of proportion between bulk and nutritiveness. The food is too concentrated, and does not contain a sufficiency of the non-nutritive matters to excite mechanically the peristaltic action of the bowels, so prone to constipation in pregnancy.

On the other hand, most articles of vegetable food, and especially the cereals, contain a sufficiency of histogenetic elements to supply even the extraordinary demands of pregnancy; while the husky coverings of the grains and the seeds of many fruits excite the action of the bowels. This is especially true of wheat, which is made up of such a happy combination as justly to entitle it to the appellation of "the staff of life."

From these facts, and others that might be adduced, the conclusion is, that vegetable diet, as a general rule, is best adapted to the condition of pregnancy. Still, no Procrustean rule should be laid down, but reason, common sense and individual peculiarities should be our guide in choosing the diet of a pregnant woman, as in all other cases. My opinion is, that a *mixed* diet is best suited to the majority of cases. As to particular articles of diet, I have not much to say. These must be adapted to the digestive powers, and to the capacities and whims of that most capricious organ, the stomach, which never shows itself more whimsical than during gestation.

In choosing the diet, special attention should be given to the torpid condition of the bowels, before alluded to. But it should be remembered, that while wheat contains all the necessary elements for calorification, nutrition, etc., that this is by no means true of *fine flour*. It is well known that fine bolted flour is highly constipating from its glutinous nature, while its constipating effect is increased by the absence of the husks which act mechanically on the bowels. It is also well known that these husks contain the inorganic or bone-making elements so necessary to maintain the integrity of the osseous structure of the mother and to develop the bony system of the new being, which must draw all its supplies from her.

The food of pregnant women, then, should consist of unbolted flour bread, corn bread, corn grits, wheaten grits, ripe fruits in season, stewed fruits, syrups, and such vegetables as are of a laxative nature. If any of these should disagree, the quantity should be first reduced; and if this does not succeed, the offending article should be sought out, and discarded from the dietary. The food should, of course, be plain and unstimulating, in accordance with the special principle announced in the beginning for our guidance in the hygienic management of pregnancy.

We now pass to a kindred subject—DRINKS. Here we have still the same great fact or principle for our guidance, viz: *That pregnancy is a state of excitement bordering on fever, and that gestation is attended with a marked exaltation and activity of almost all the mental and bodily functions.* This is especially true of the nervous system, which is preëminently impressible, and ready to respond, in an exaggerated and abnormal manner, to every exciting agent, whether direct or reflex, whether mental or physical. If, then, high-seasoned, stimulating and over-abundant food should be eschewed in pregnancy, what shall we say of those too common beverages and stimulants—tea, coffee, and alcohol? While the injurious effects arising from food improper in quality or excessive in quantity are to a great extent localized, acting mostly through the sympathies of the stomach, there is a large class of drugs—such as opium, hyoscyamus, prussic acid, etc.—that act specifically, if not directly, on the nervous system, exciting or obtunding its sensibilities. In this class, I have no hesitation in placing tea and coffee.

As to alcohol, it is universally admitted that it produces a morbid condition of the body generally, and of the nervous system specially. It is also conceded that alcohol acts injuriously, even in moderate quantities, "by obstructing the removal of the hydro-carbonaceous products of the continual disintegration of the tissues, in virtue of the stronger affinity which it has for oxygen, whereby it will prevent the respiratory process, from exerting its due influence on the purification of the blood."—*Carpenter.*

Certainly, then, alcoholic drinks should not be indulged in during pregnancy, when pure blood, quiet nerves, equanimity of mind, and a moderate circulation, are absolutely essential to the health of both mother and offspring.

But while all will admit this with regard to alcohol, some may not be so ready to reject tea and coffee. Yet it cannot be denied that tea and coffee contain little or no nutriment, and that tea, and especially green tea, is astringent; while both tea and coffee are particularly exciting to the brain and nerves, producing effects very similar to the narcotic drugs. Even admitting that tea and coffee dimish the waste of nitrogenized tissues, as some physiologists contend, the checking of this waste is but a poor compensation for the nervous excitement that must result from the use of such beverages in pregnancy, with its excessive nervous excitability, which is ever ready to manifest itself in the most distressing and dangerous disorders.

And again, as to this waste of the mother's tissues, it may, in many cases, be necessary to the nourishment of the child she bears, when the external supplies are insufficient; and even though such abstraction of the nutritive elements of the mother may not be essential to the development of the *fœtus*, it were better for the mother to become as lank and lean as any hungry Cassius, than to have her nervous system deranged by such beverages as tea and coffee.

Finally, discarding all theoretical or physiological considerations, we have abundant proofs of the injurious effects of tea and coffee in the sallow complexions and in the multiform nervous and dyspeptic derangements everywhere to be seen as results of the excessive use of these beverages.

The conclusion, then, is, that the only proper beverages for pregnant women are pure water and milk. This being true, it should be insisted on in our hygienic management of this condition; and we should do all that we can to counteract the growing demand not only for tea and coffee, but for lager-beer, porter, ale, wine, and all kinds of villainous and ruinous alcoholic liquors. Let enlightened physicians put their ban on all such things, which are destructive alike to health and good morals.

The next subject to which I invite attention is that of VENTILATION and INSOLATION.

It is sufficient to say, on this point, that pure air, so essential to health in all cases, is especially so during the act of gestation. While proper food is necessary for the nutrition of mother and offspring, pure air is equally needful to decarbonize the blood, and to keep it in that pure vitalized condition which will make it suitable for the health of the mother and for the development of the child. The necessity for a pure condensed and highly-oxygenated air is especially great in the latter stages of pregnancy, when the diaphragm is pushed upward by the ascending womb—when the capacity of the lungs is diminished—when the liver and great blood-vessels are compressed, the circulation oppressed, and the secretions checked or greatly retarded. Under such circumstances as these, confinement in-doors to a close, heated, rarified and desoxygenated air must inevitably poison the whole circulating fluid, derange the functions of every organ, and exert the most disastrous influence on mother and child. Without going into details as to the various diseases that may be thus superinduced, I will only say, that impurity of the mother's blood, from the causes above mentioned, and from many other sources, is doubtless one of the most frequent causes of abortion, and of the death of the fœtus in utero; and it is not at all unreasonable to presume, that from the same cause originate many cases of deformity, and especially of that large class dependent on *arrest of development.*

The apparently well-established action of chlorate of potash in preventing abortion by its vivifying, oxygenizing action on the blood, would seem to confirm the correctness of these views. But we are not left to draw our conclusions on this interesting point from doubtful therapeutical observations as to the action of a particular remedy; for reason, general experience, the teachings of chemistry, physiology and pathology all demonstrate, conclusively, that impure blood, in gestation, is the most influential cause of the grave diseases and accidents which befall both mother and child. It is equally well established, that impure blood is oftener the result of impure and insufficient air than anything else, unless improper food and drinks be excepted. It is also true, that the great remedy for impure blood, originating from impure or insufficient air, is to be sought, not in chlorate of potash or any other *drug*, but in an abundance of *pure air.*

We will next consider LIGHT as a hygienic agent in gestation.

To appreciate the effects of light on organized bodies, it is only necessary to observe those plants that grow up in the shade. Plants thus excluded from light are colorless, sickly, and brittle. It is well known that light has the power of reversing completely the vital and chemical changes of vegetable life; for vegetation absorbs carbon and gives out oxygen in the day, while at night just the reverse is true. It is not at all unreasonable to suppose that a similar and even as great control is exerted over animal life by the combined action of light and heat. But we are not left to supposition in this matter: the sallow, bloodless faces and the feeble bodies of the denizens of narrow streets and alleys and dark cellars, and the anæmic appearance of the secluded drawing-room belle, all demonstrate the identity of the animal and vegetable kingdoms in their relations to heat and light. While this identity doubtless exists, it has attracted less attention than its importance demands, because the absence of light has almost always been associated with bad air, unwholesome food, filth, and other circumstances which have been considered sufficient in themselves to account for ill-health.

It being admitted, then, that light and heat have a powerful influence in decomposing and dissipating poisonous emanations, whether animal or vegetable—it being also granted that they give color and firmness to the skin, and greatly promote its important eliminative and absorptive functions, while they are essential to perfection of form and full physical, development—it follows that these agents, light and heat should be highly regarded in the hygiene of gestation.

We should insist on it, then, that pregnant women under our charge should be much exposed to the heat of the sun and the light of *day;* for we have no reason to believe that artificial light has any sanative powers. The expected good is to be derived from the combined chemical and physical action of light and heat as they exist in the compound luminous and calorific rays emanating from the sun.

It may be known to some, that in addition to the "water-cure," the "movement cure," and many other cures of these modern days, that the "sun cure," or insolation, has been highly recommended. The patient, stripped, is exposed to the rays of the sun, as these are poured through an open window. The suggestion is a good one, and we should profit by it, regardless of the source.

Next, we consider one of the most important of the hygienic agents.

It is hardly necessary to tell a body of intelligent physicians that EXERCISE is absolutely essential to the proper performance of all those bodily functions on which health, and even life itself, depends. It is well known, that though respiration, circulation and digestion are involuntary, yet that the full and proper performance of these functions depends greatly on our voluntary movements. Thus, neglect of exercise weakens and disorders the stomach, and all healthful supplies are cut off;—it reduces the capacity of the chest, and thus the blood is not properly vitalized;—it prevents free circulation in the capillary vessels, and thus assimilation and all the great vital changes which should be effected in the capillary system are suspended or imperfectly performed; the muscles shrink and lose their strength, or even become paralyzed; the blood-vessels become obliterated, and cease to convey the vital fluid; the brain is torpid, the nerves sluggish or unstrung, and inordinately excitable; and, in short, universal derangement of the whole animal economy ensues, ending in stagnation or cessation of motion, which is only another name for *death* itself. Life is *motion*—constant, ceaseless motion. *Exercise* promotes all the vital movements, and stands in direct antagonism to disease and that torpor which merges

> "In the deep stillness of that dreamless state
> Of sleep that knows no waking joys again."

The conclusion, then, is, that every hygienic precept as to air, food, drink, clothing, and *everything* else, may be diligently observed; and yet if the *one thing needful*—EXERCISE—be neglected, neither body nor mind can attain its full and perfect development.

Having premised these incontrovertible facts, it only remains to apply them to the condition of pregnancy. This condition being one of excitement, as stated in the outset, it may be objected, that exercise may unduly increase this excitement, and thus be productive of bad results. But it must be remembered, that violent, exhausting or purturbating exercise is not recommended, but exercise of a moderate, healthful kind, adapted to the peculiar condition and circumstances of the woman. Of course, excessive exertion—straining, lifting, jolting, and all violent gymnastics—should be avoided. Such exercise as this—walking every day in the open air, with the shoulders well thrown back and the chest well expanded, carriage riding, or even horseback riding on an easy-going horse—will not only be free from injurious consequences, but will be highly conducive to health, by promoting the proper performance of all the bodily functions, and especially by increasing the activity of the skin, lungs, and other depurating organs, which is so necessary to keep the blood pure, that it may supply suitable pabulum to preserve the health of the mother and to develop her offspring. And even admitting that the exercise may be sufficient to increase the force and frequency of the heart's action, this temporary excitement will be more than counterbalanced by the bracing, invigorating and equalizing effect on the nervous system, the cheerfulness of mind, and the buoyancy and hopefulness of feeling resulting from such exercise.

We cannot, then, too strongly insist on proper exercise during gestation. And it is *necessary* for us to do this; for though women in the lower walks of life suffer from inordinate compulsory exertion, yet women of the "higher classes" suffer even more from insufficient exercise, from indolence, and confinement in close rooms, than from any other single cause.

After exercise, the subject of rest or SLEEP properly comes up for consideration. But I will only say, on this point, that the amount of sleep necessary for human beings varies according to age, habits of life, and probably according to sex. It is fair to presume, from the peculiar organization of woman, that she needs more sleep than man. And this is undoubtedly true of pregnant women. The great excitability of the nervous and vascular systems, already so much insisted on as the basis of all our hygienic measures in pregnancy—the extraordinary drafts made on the mother during gestation—urgently demand an extra amount of sleep. But while this is true beyond a doubt—while all nature proclaims that night was "for rest ordained and soft repose"—while even the lower animals, obedient to the signal, retire to their "grassy couch" when night spreads her sable curtain—yet multitudes of reasoning, intelligent men and women make night a time for soul-and-body-destroying dissipations and "teeming mischiefs." This is the time for fashionable parties, where women most do congregate, and where, in addition to want of rest, the system is poisoned by impure air, and oppressed by excessive and improper eating and drinking; while the mind and all the moral feelings are worked up into a feverish state of excitement, which reacts with terrible effect on the poor, abused, over-excited and over-burdened physical frame. And alas! among these senseless devotees of fashion are found women bearing within them a being whose mental, moral and physical well-being is dependent on their observance of the laws of health.

Much might be said, in this connection, with regard to the mental hygiene of gestation; but I can only say, under this head, that women, and especially *pregnant* women, should avoid purturbating and exciting moral influences, as far as possible. This teaching comports with the general peculiarities of her organization, and also with the special condition of excitement in which she is placed by reason of gestation. As to her physical organization, it is sufficient to say that it is naturally more delicate and susceptible than that of man, while this natural susceptibility has been much increased by the enervating habits of civilized life; her feelings are naturally acute and impressible, and this inherent mental condition has been nursed into a morbid sensitiveness under the unnatural social influences by which she is surrounded. Hence, her feelings are easily excited, and they act with terrible energy on her feeble and sensitive body; and the latter reacting on the former, such a commotion is often produced that the frail tabernacle in which she dwells is prostrated and destroyed.

In view of these facts, I may add, that of all human beings, women, and especially child-bearing women, are under the greatest, the most sacred obligation to obey all the laws of health, and especially the laws of mental hygiene.

The CLOTHING of pregnant women should, of course, be perfectly loose, and corsets, whalebones, and everything of the kind should be banished just as soon as there is a reasonable presumption that pregnancy may exist. All physicians have witnessed the evils arising from the foolish attempts of women to conceal their condition; it is needless to enumerate them.

The feet and legs should be protected by thick shoes and drawers. Garters should be dispensed with, especially in the latter months. Night-caps should also be banished, as they keep the head unduly warm.

The POSITION of pregnant women is worthy the attention of physicians. Women in this condition should be cautioned against stooping forward, so as to compress the abdomen, and they should be advised to avoid all constrained and unnatural postures.

BATHING should never be neglected in pregnancy. To subdue the nervous and vascular excitement incident to this condition, and to depurate the blood, there is nothing so safe and effectual as tepid, cool, or even cold baths. These baths abstract excessive heat, equalize the circulation, remove internal congestions, and strengthen the whole system, thus preparing it to furnish suitable elements for the new being, and to pass safely through the critical time of parturition. The feelings of the patient may be safely consulted as to the temperature of the water. The sponge-bath or wash-down is the best mode of applying it. Of course, the shower-bath, and all applications that cause a violent shock, should be avoided. Pregnant women generally bear cold water remarkably well, but they cannot well tolerate violent impressions of any kind. The sponge-bath should be used every day, or every other day, from the beginning of pregnancy, and the hip-bath should be resorted to once a day during the last month or two. The temperature of this should be moderate, and it should not be continued more than five or ten minutes each time. I have advised the above course in a number of cases, with the most happy results. The cold hip-bath, in connection with the general baths recommended, places the system in the most favorable condition for parturition; and my experience teaches me that the pains of labor may be almost entirely prevented by a proper hygienic preparatory course, of which I consider bathing a most important part.

I might give many examples of the advantages of such a course as the one here recommended, in preventing the ills of pregnancy and the pains of labor. I extract from a publication made by me before the war:

A lady under my charge is now pregnant with her fifth child, and is near her confinement. In her previous pregnancies, she lived *after the general manner of women*, but still much better than many. She has heretofore had almost all the disagreeable symptoms of pregnancy, which have been much aggravated by ulceration of the womb. She has suffered from palpitation of the heart, most distressing heart-burn, costive bowels at one time, and the most violent straining and bearing-down dysentery at another; she has had headache, swelled legs, cramps in the stomach, and many other symptoms too tedious to mention. In her present pregnancy, she has followed the wholesome directions above given, and she has had scarcely a disagreeable symptom, up to this time, except morning sickness in the early part of her pregnancy. It is now only a week or two to her confinement, and her bowels have been regular all the time; she has never been troubled with sour stomach or heart-burn; her legs are not the least swelled; and, in short, she is vigorous, lively, active, hopeful, and in the enjoyment of perfect health.

To this report I may add, that in due time the subject of it was delivered in about three hours, with only two or three *painless* "pains," when previous labors had been protracted to ten or twelve hours, with no little pain. I may also say, that I have had several cases of this kind, and that I am firmly convinced, both by reason and observation, that parturition, in a favorable normal condition of the system, is not *necessarily* a painful process; and I feel no hesitation in saying that labor may, in many cases, be rendered almost entirely painless by those hygienic agents, which will place the system in the highest state of health, and remove that morbid irritability and that excessive rigidity superinduced by bad habits of living.

I might dilate on this subject by giving additional reasons for this belief; but enough has been said to call attention to this most important and interesting point; and I fear that I have already too greatly protracted this article, while at the same time I feel that the subject merits even more extended consideration.

---

Cure of Aneurism by Flexion.—Dr. B. L. Hovey, in a paper read before the Monroe County Medical Society, New York, gave a detailed history of a case of traumatic aneurism, situated in the scarpal triange, and caused by a buckshot. The patient endured compression of the artery as it passes over the pubic bone for twelve days, until ulceration of the integument took place, when it was deemed advisable to desist further treatment by this process. The thigh was then flexed upon the body, with a well-adjusted pad between. The result of the treatment was success. The increasing and hard pulsating tumor had diminished in size one-half, and was nearly solid, and only a slight thrill was distinguished by the touch.

